# Distribution, Genesis, and Human Health Risks of Groundwater Heavy Metals Impacted by the Typical Setting of Songnen Plain of NE China

**DOI:** 10.3390/ijerph19063571

**Published:** 2022-03-17

**Authors:** Yuanzheng Zhai, Fuxin Zheng, Dongfan Li, Xinyi Cao, Yanguo Teng

**Affiliations:** Engineering Research Center for Groundwater Pollution Control and Remediation of Ministry of Education of China, College of Water Sciences, Beijing Normal University, Beijing 100875, China; zyz@bnu.edu.cn (Y.Z.); 202131470011@mail.bnu.edu.cn (F.Z.); 201821470013@mail.bnu.edu.cn (D.L.); 202021470003@mail.bnu.edu.cn (X.C.)

**Keywords:** groundwater, heavy metals, correlation analysis, human health risks, Songnen Plain

## Abstract

Heavy metals pollution in groundwater and the resulting health risks have always been an environmental research hotspot. However, the available information regarding this topic and associated methods is still limited. This study collected 98 groundwater samples from a typical agricultural area of Songnen Plain in different seasons. The pollution status and sources of ten heavy metals (As, Ba, Cd, Co, Cr (VI), Cu, Fe, Mn, Ni, Pb, and Zn) were then analyzed and compared. In addition, the human health risks assessment (HHRA) model was used to calculate human health risks caused by heavy metals in groundwater. The results revealed that heavy metals were mainly distributed in the northwest of the study area and along the upper reaches of the Lalin river and that the concentrations of heavy metals were higher during the wet season than the dry season. Industrial and agricultural activities and natural leaching are the main sources, and each kind of heavy metal may have different sources. Fe and Mn are the primary pollutants, mainly caused by the native environment and agricultural activities. The exceeding standard rates are 71.74% and 61.54%, respectively based on the Class III of Quality Standard for Groundwater of China (GB/T 14848-2017). The maximum exceeding multiple are 91.45 and 32.05, respectively. The health risks of heavy metals borne by different groups of people were as follows: child > elder > young > adult. Carcinogenic heavy metals contribute to the main risks, and the largest risks sources are Cr and As. Therefore, the government should appropriately restrict the use of pesticides and fertilizers, strictly manage the discharge of enterprises, and control man-made heavy metals from the source. In addition, centralized water supply and treatment facilities shall be established to prevent the harm of native heavy metals.

## 1. Introduction

Groundwater pollution with heavy metals, which are metals that have densities > 5 g/cm^3^, is a complex and urgent problem [1,2]. In agricultural areas, heavy metals enter the soil when fertilizer, pesticides, and other agricultural production materials are used in large quantities [3]. Because of their recalcitrant nature and ability to bioaccumulate, the heavy metals content of the soil will be maintained at a high level following contamination [4]. Heavy metals in the soil can enter groundwater through leaching and osmosis. Excessive heavy metals can harm the environment and human health [5]. Agricultural activities are one of the main sources of heavy metals pollution in groundwater [6,7]. Pesticides and fertilizers are often abused as a result of imperfect environmental management systems, especially in developing countries, to ensure adequate food supplies [8,9,10]. Heavy metals often enter aquifers with pesticides, fertilizers, and livestock manure, causing a direct threat to human health [11,12].

Songnen Plain is a major agricultural area in China with a long history of soil and groundwater pollution caused by agricultural activities [13]. Unlike agricultural areas commonly found in Europe and the United States, Songnen Plain is in the industrial base of Northeast China, where there is a dense population and complex environment. As a result, human activities and industrial production in this area further increase the risk of groundwater pollution while also bringing challenges to conducting studies in the area [14]. Despite these challenges, many studies have investigated groundwater pollution and the associated risks in this area. Studies have ever analyzed causes of deterioration of the groundwater quality and health risks posed by typical pollutants in Songnen Plain [15,16,17]. However, most of the work focused on typical pollutants with the largest concentration and widest distribution, like trinitrogen, Fe, Mn, and so on. As a result, their findings were representative to an extent, but not comprehensive. Indeed, the toxicity of some heavy metals (such as As and Cr) can threaten human health at a low concentration [18]; therefore, it is necessary to carry out a comprehensive health risks assessment of heavy metals in this area. The human health risks assessment (HHRA) method is a model recommended by the United States Environmental Protection Agency (USEPA) which combines the degree of water pollution with human health to evaluate the harm caused to humans by water pollution [19,20]. This method has been widely used in groundwater health risk assessments [21,22,23]. The model usually considered two health risks categories: intake (generally through eating and drinking) and contact (generally through skin and breathing) [19,20]. Zhai et al. (2017) [24] evaluated the non-metallic pollutants risks of groundwater in the Songnen Plain by using this model. The results successfully reflected the health risk status of the typical pollutants such as trinitrogen. However, unlike air and dust, most people are exposed to water for a very short time every day, the risk posed by direct contact with the groundwater such as the skin exposure in the shower is generally less than 1% of the total. [25,26]. Therefore, compared with the previous studies, this paper pays more attention to the health risks from drinking. This not only reduces the unnecessary calculation process, but also reduces the model uncertainty caused by the evaluation of exposure parameters. On the basis of finding out the distribution and source of heavy metals, the HHRA model can quantitatively analyze the health risks value generated by each metal and evaluate the health risks of the whole region.

Therefore, the present study was conducted to investigate groundwater heavy metals pollution in agricultural areas of Songnen Plain and quantitatively evaluate the health risks posed by heavy metals in groundwater. To accomplish this, a traditional industry and agriculture area focusing on the following aspects was selected: 1. the distribution characteristics of 11 heavy metals (As, Ba, Cd, Co, Cr(VI), Cu, Fe, Mn, Ni, Pb, and Zn) in groundwater of typical agricultural areas in Wuchang city were determined through sampling and data collection during different seasons and their pollution status was discussed. 2. Based on the Spearman correlation analysis and Principal component analysis (PCA), the genesis of heavy metals in the groundwater of Wuchang city was calculated and their sources were identified. 3. Health risks of heavy metals in groundwater to different groups of people were evaluated. Additionally, the present study also compares the differences in health risks caused by heavy metals and non-metallic metals pollution. Although some elements have been discussed as typical pollutants in previous studies, the comprehensive heavy metals investigation and assessment has not been considered before. The research results can be used to help environmental managers establish corresponding treatment and control measures to protect groundwater and human health.

## 2. Materials and Methods

### 2.1. Study Area

**Figure 1:**
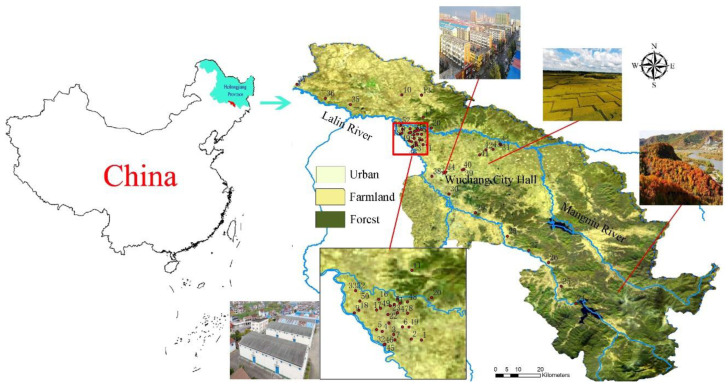
Location of the study area and sampling points.

The study area is located in the riverside source field of Wuchang, in northeastern China, at 126°58′ and 127°07′ E longitude and 45°02′ and 45°05′ N latitude, covering approximately 100 km^2^. This area, located at the intersection of two rivers, is bound to the west by the Lalin River and the east by the Mangniu River (Figure 1). A large area of rice is planted in the area, and the north is mountainous forest land. The urban area is mainly within five kilometers of the Wuchang City Hall. At the intersection of the Lalin River and the Mangniu River, there are a large number of villages, animal husbandry farms, and small factories, which mainly engaged in grain, animal husbandry product processing, and metal processing. This is also the key area of the sampling (Figure 1).

The study area is characterized by a temperate continental monsoon climate which the mean annual temperature of approximately is 3.6 °C and summer maximum and winter minimum temperatures of 36.5 °C and −40.9 °C, respectively. The mean annual precipitation is approximately 619.7 mm, with most (65–70%) occurring from June to August. The mean annual evaporation is approximately 1321.4 mm, and the mean annual number of frost-free days is approximately 179 days. The above data were collected from the local meteorological and water conservancy departments. The primary aquifers in the study area are composed of Quaternary sediments with a thickness of 30–60 m, which act as the main groundwater storage system. The sediments are composed of sandstone, pebbly medium-coarse sandstones, sand gravel, and gravel pebbles [14,15,21]. Based on the lithological properties, geological age, distribution of aquifers and aquitards, and hydrodynamic conditions, the Quaternary sediments can be divided into a submersible water aquifer group and a confined water aquifer group. The submersible water aquifer, which consists of fine sandstones, coarse sandstones, and sand gravel, thickens gradually from south to north and has a thickness between 37.5 m and 51 m. Vertically, the grain size changes from fine in the top to coarse in the bottom. Horizontally, the grain size changes slightly from east to west and varies from coarse to fine near the Lalin River. For the confined water aquifer, which is composed of fine sandstones, coarse sandstones, and sand gravel, the thickness is between 26.5 m and 31 m. Vertically, this aquifer has the same regulation as the submersible water aquifer and no apparent change in the horizontal direction. The aquifers are recharged by the infiltration of precipitation, lateral flows, and agricultural irrigation infiltration, while discharge mainly occurs via artificial exploitation [14,15,21].

### 2.2. Sampling and Analysis

The toxicological indexes of 11 heavy metals (As, Ba, Cd, Co, Cr(VI), Cu, Fe, Mn, Ni, Pb, and Zn) were selected for investigation in this study. The assessment of water quality was subject to the national standard: Class III of Quality Standard for Groundwater of China (GB/T 14848-2017). A total of 98 groundwater sampling points were arranged to collect, including 52 in the wet season (Nos. 1–52) and 46 in the dry season (in the same positions as the wet season points except for Nos. 4, 5, 7, 14, 16 and 17). The groundwater samples were collected from a shallow unconfined aquifer. Considering that the ultimate goal of this study is to serve human health, the sampling points were set in densely populated areas rather than evenly distributed. In addition, sampling points were also arranged along the Lalin River, which is one of the main recharges of the groundwater in the study area. The analysis was performed in our laboratory. Fe, Mn, and Ba in the samples were determined by using a plasma spectrometer (ICP-AES), and the instrument used was Agilent 4210 MP-AES. As, Cd, Co, Cr(VI), Cu, Ni, Pb, and Zn were determined by using a plasma mass spectrometer (ICP-MS), and the instrument used was Agilent 7700 (Table S1).

To ensure the comparability, accuracy, and representativeness of the samples, quality assurance, and quality control were applied in the processes of both sampling and laboratory analysis stage based on the Technical Specifications for Environmental Monitoring of Groundwater (China, HJ/T 164-2020). In the sampling stage, all sampling personnel were trained before they got their sampling certifications. Each groundwater well was taken at least 20 min after pumping commenced to ensure the stability of water quality. Field parallel samples and full procedure blank samples as well as groundwater samples were collected together and were expedited to the testing institution immediately after sampling to minimize storage time. All samples during the monitoring period were sent to the same testing institution which has the certificate of the China Metrology Accreditation. The standard correlation coefficients curves of all metals were above 0.999, the relative standard deviation was less than 10%, the recovery efficiencies were between 85–120%, and the relative error was less than 10% (Table S1). In the testing stage, all instruments and gauges were certified by the Metrological Department or calibrated by analysts, and all the monitoring and analysis methods used were taken from the national standard (or recommended) methods of China.

### 2.3. Methods

#### 2.3.1. Source Identification Methods

Heavy metals in groundwater come from various sources, and a kind of heavy metal may have various sources. The correlation analysis between heavy metals in groundwater can reveal the sources of heavy metals effectively [27]. In this study, Spearman correlation was performed to calculate the significance and correlation coefficient. Spearman correlation analysis is widely used in the data of non-normal distribution hydrochemical processes, and the linear correlation between two parameters can be evaluated. The closer the correlation coefficient is to 1, the stronger the correlation between the two parameters, and the more likely they are to have the same source [28]. Principal component analysis (PCA) is a statistical method that can simplify and merge several original variables through dimension reduction. Furthermore, it extracts several comprehensive variables and factors to reflect the common characteristics among research objects [24]. The methods can ensure maximum accuracy under the least loss of information. In this study, Spearman correlation analysis and PCA were performed by using the IBM SPSS 26.

#### 2.3.2. Model for Evaluation of Human Health Risk

The HHRA consists of a carcinogenic pollutant (As, Cd, Cr(VI)) evaluation model and a non-carcinogenic pollutant (Ba, Co, Cu, Fe, Mn, Ni, Pb, Zn) evaluation model. This study considered four groups of the target population: child (0–8 years old), young (9–17 years old), adult (18–60 years old), and elder (>60 years old) [29]. In this model, the average carcinogenic risk (R) was generally used to assess the potential health risks of heavy metal intake via groundwater and the limit for R value stipulated by the USEPA (1 × 10^−4^ a^−1^) was adopted as the reference standard, in a unit of a^−1^. Parameters of the HHRA model are shown in Table S2. Specifically, groundwater was sampled during both the wet and dry seasons, and the calculation results have been discussed respectively. The intake per unit weight was used to describe the heavy metals toxicity [30].

The human health risks assessment model of carcinogenic heavy metals in groundwater [31] was as follows:

(1) $$R_{i}^{c}=\frac{1-\exp(-D_{i}q_{i})}{L}$$

where $R_{i}^{c}$ is the health risk posed by a carcinogenic heavy metal *i* entering individuals through drinking water, a^−1^; *D_i_* is the average daily exposure dose per unit body weight of a carcinogenic heavy metal *i* entering individuals through drinking water, mg/(kg·d); *q_i_* is the coefficient of a carcinogenic heavy metal *i* entering individuals through drinking water; and *L* is the life expectancy of Chinese individuals, a.

The human health risks assessment model of non-carcinogenic heavy metals in groundwater [31] was as follows:

(2) $$R_{j}^{n}=\frac{D_{j}\times 10^{-6}}{R_{fDj}L}$$

where $R_{j}^{n}$ is the health risk posed by a non-carcinogenic heavy metal *j* entering individuals through drinking water, a^−1^; *D_j_* is the average daily exposure dose per unit body weight of a non-carcinogenic heavy metal *j* entering individuals through drinking water, mg/(kg·d); *R_fDj_* is the average daily reference dose per unit body weight of a non-carcinogenic heavy metal *j* entering individuals through drinking water, mg/(kg·d); and *L* is the life expectancy of Chinese individuals, a.

*D_i_* and *D_j_* were calculated by using the following formula:

(3) $$D_{i/j}=K\rho_{i/j}$$

where *K* is the per capita drinking water intake per unit weight, L/(kg·d); and *ρ_i/j_* is the measured mass concentration of carcinogenic and non-carcinogenic heavy metals.

The health risks hazard value *R* caused by heavy metals in groundwater was the sum of the carcinogenic heavy metal risks value *R_c_* and non-carcinogenic heavy metal risks value *R_n_*:

(4) $$R_{c}=\sum_{i=1}^{l}R_{i}^{c}$$

(5) $$R_{n}=\sum_{j=1}^{k}R_{j}^{n}$$

*R = R_c_ + R_n_* (6)

## 3. Results

### 3.1. Temporal and Spatial Distribution of Heavy Metals

**Figure 2:**
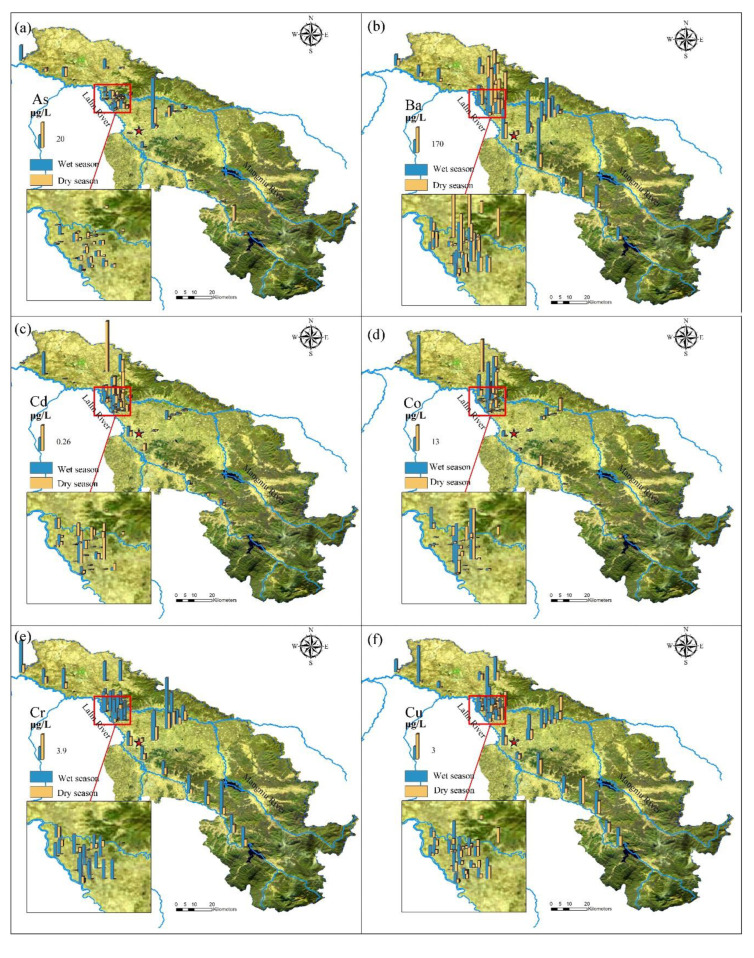

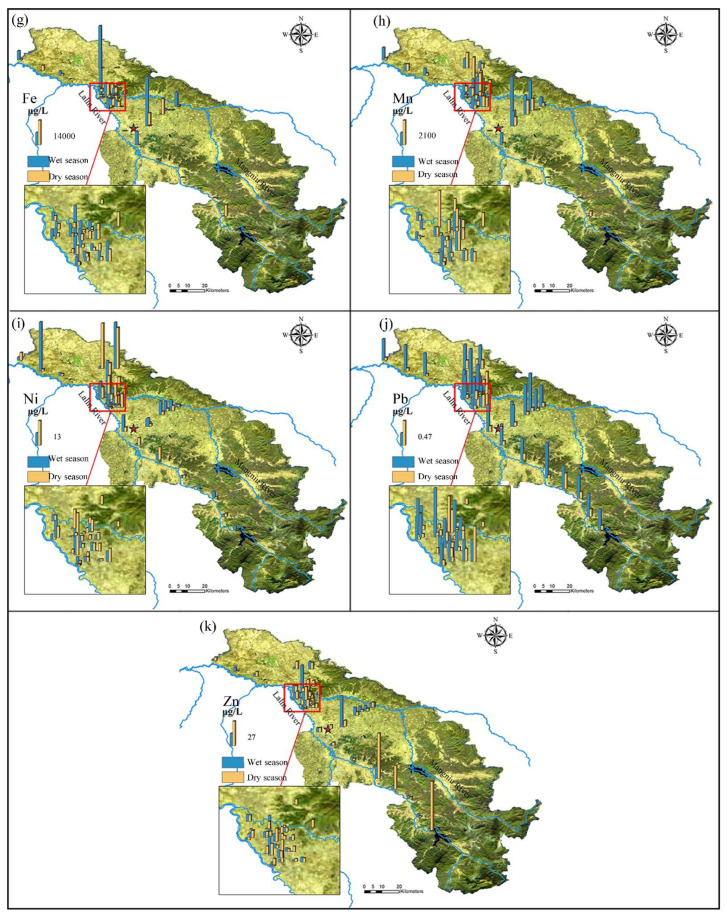
Spatial distribution of concentrations of the heavy metals: (**a**) As, (**b**) Ba, (**c**) Cd, (**d**) Co, (**e**) Cr, (**f**) Cu, (**g**) Fe, (**h**) Mn, (**i**) Ni, (**j**) Pb, (**k**) Zn.

The spatial distribution of groundwater concentrations in the study area differed greatly between the wet and dry seasons (Figure 2), with the concentrations in the former being obviously higher on the whole. Among carcinogenic pollutants, the concentrations of As and Cd showed high levels in the densely populated area (Figure 2a,c). Cr showed a high concentration in the whole sampling points in the wet season while decreased significantly in the dry season (Figure 2e). The highest As and Cr showed in the middle part of the study area while the lower in the north part of the study area. Among non-carcinogenic pollutants, the concentrations of Ba, Co, Cu, Fe, Mn, and Pb showed higher levels in the wet season than the dry season, while the highest concentrations of Ni and Zn in the dry season were higher than in the wet season (Figure 2i,k). Non-carcinogenic pollutants were mostly distributed in densely populated areas. Among them, the concentration of Co was high at some points in the urban area (Figure 2d), while others showed a low concentration. What’s more, Ba, Cu, and Pb also showed a high concentration along the Lalin River (Figure 2b,f,j). Compared with people and factories distribution, concentrations near the river showed stronger seasonal differences, that is, the concentration in the wet season was much higher than that in the dry season. The temporal distribution of Zn along the river was just the opposite, and it was higher in the dry season (Figure 2k).

### 3.2. Statistical Characteristics of Heavy Metals

Groundwater heavy metals concentrations were higher in the wet season than in the dry season expect for Cd (Table 1). In the wet season, the average concentration of heavy metals: Fe (2779.91 μg/L) > Mn (817.73 μg/L) > Ba (110.89 μg/L) > Zn (8.11 μg/L) > Ni (6.87 μg/L) > Co (3.23 μg/L) > As (3.12 μg/L) > Cr (2.98 μg/L) > Cu (1.75 μg/L) > Pb (0.48 μg/L) > Cd (0.05 μg/L). The specific range of concentrations were as follows: 0.24 to 39.78 µg/L for As, 41.20 to 337.60 µg/L for Ba, 0 to 0.32 µg/L for Cd, 0.09 to 26.32 for Co, 1.96 to 7.80 µg/L for Cr, 0.67 to 5.93 µg/L for Cu, 8.69 to 27,434.20 µg/L for Fe, 1.00 to 4258.09 µg/L for Mn, 0.37 to 26.65 µg/L for Ni, 0.40 to 0.95 µg/L for Pb and 1.27 to 54.18 µg/L for Zn. While in the dry season, Fe was also the highest average concentration metal: Fe (1501.62 μg/L) > Mn (609.08) > Ba (78.53) > Zn (7.84) > Ni (3.85) > Co (2.53) > As (2.18) > Cu (0.76) > Cr (0.63) > Cd (0.52) > Pb (0.14). The specific range of concentrations were as follows: 0.16 to 15.63 µg/L for As, 9.78 to 348.29 µg/L for Ba, not detected for Cd, 0.03 to 26.64 for Co, 0 to 2.36 µg/L for Cr, 0.12 to 3.10 µg/L for Cu, 5.62 to 8754.59 µg/L for Fe, 0 to 3205.13 µg/L for Mn, 0.02 to 24.50 µg/L for Ni, 0.03 to 0.72 µg/L for Pb and 0.31 to 54.29 µg/L for Zn.

From the whole, standard deviation (SD) values in the wet season were bigger than in the dry season and most of the metals showed an uneven distribution except for Pb and Cd. Especially for Fe and Mn, had a huge spatial difference with a number of 5559.29, 609.68 in the wet season and 2605.05, 722,24 in the dry season, respectively. Variation coefficient (VC) values were all at a low level, and the gap was small among different metals.

Four kinds of metals showed excessive signs, both exceedance rate (E) and maximum exceedance multiple (MEM) in the wet season were higher than those in the dry season. The most serious metal was Fe and Mn, which had an E value of 42.31% and 71.74% in the wet season and 39.13% and 61.54% in the dry season. The MEM of Fe and Mn was 91.45 and 42.58 in the wet season and 29.18 and 32.05 in the dry season. As and Ni also exceed the standard, but the degree was lighter than the formers.

### 3.3. Multivariate Statistical Analysis

#### 3.3.1. Correlation between Heavy Metals

In the wet season, at the level of 0.01, NO_3_^-^ and Cl^-^ showed a significant correlation, the correlation coefficient was 0.77. Cd, Cu, Fe, Mn, and Ni showed a significant correlation with NO_3_^-^ or Cl^-^. Cd, Co, and Ni showed a significant correlation in Spearman correlation analysis results between each other (Table 2), and the correlation coefficients were 0.691, 0.680, and 0.735, respectively. Fe, Mn, and As had the same situation, 0.627, 0.603, and 0.649, respectively. Ba-Co and Cr-As showed a weak significant correlation, the correlation coefficients were 0.352 and 0.462, respectively. Cu showed a significant correlation with Ni (0.493) and a significant negative correlation with Fe (−0.513). Zn showed a significant correlation with Pb and Cd (0.409 and 0.492, respectively) and a significant negative correlation with As (−0.378).

In the dry season, at the level of 0.01, NO_3_^−^ and Cl^−^ showed a significant correlation with a value of 0.77. Cd, Cu, Fe, and Ni were significantly correlated with NO_3_^−^ or Cl^−^. Cd, Co, and Ni showed a significant correlation, and the correlation coefficients were 0.419, 0.358, and 0.611, respectively. Fe, Mn, and As had the same situation, 0.634, 0.702, and 0.612, respectively. Cu, Cd, and Pb showed a weak significant correlation, and the correlation coefficients were 0.363, 0.417, and 0.395, respectively. Ba showed a weak significant correlation with Mn and Ni, the correlation coefficients were 0.394 and 0.455, respectively. Cr showed a negative significant correlation with Ba, Ni, and Zn, the correlation coefficients were −0.511, −0.492, and −0.369, respectively.

#### 3.3.2. Principal Component Analysis (PCA)

Before the principal component analysis, the data were tested by Kaiser-Meyer-Olkin (KMO) and Bartley sphere tests to ensure that the data can be used in the PCA. The common factors were extracted by the principal component method, and the component matrix was rotated by the maximum variance method. A total of four common factors with eigenvalues above 1 were extracted: F1, F2, F3, and F4, and their cumulative variance contribution rate was 69.419% (Table 3, Figure S1).

### 3.4. Potential Human Health Risks

**Figure 3:**
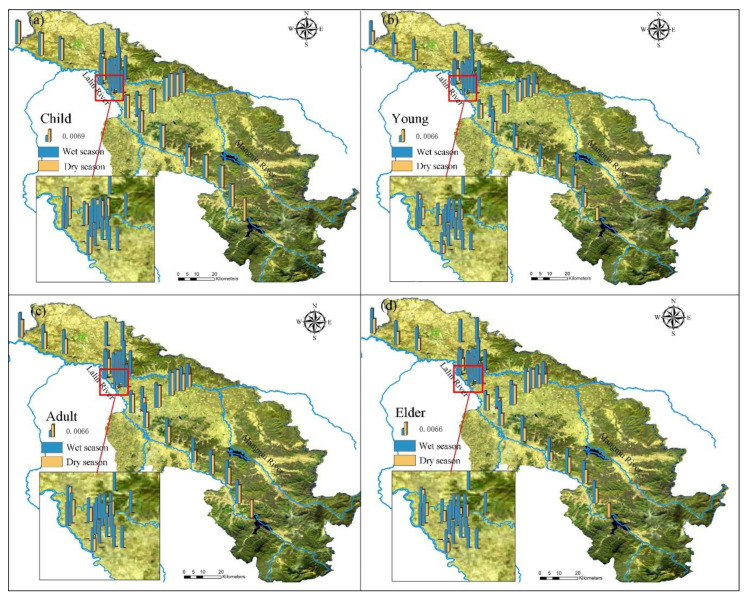
Spatial distribution of R-values: (**a**) child, (**b**) young, (**c**) adult, (**d**) elder.

Based on the HHRA results (Figure 3), the health risks varied seasonally and with age, which occurred in the following order: child> elder > young > adult. During the wet season, the average R value of a child, elder, young, and adult was 0.0132, 0.0124, 0.0111, and 0.0110 a^−1^, respectively, while in the dry season 0.0070, 0.0050, 0.0040, and 0.0039 a^−1^, respectively (Figure S2). However, there was consistency in the overall health risks, that is, the R values for all of the sampling points were far higher than the limits (0.0001 a^−1^), the high values occurred along the upper reaches of the Lalin river. In the dry season, health risks of 23 sampling points were acceptable, which were mainly distributed in the middle of the study area. Other points were higher than the reference value, and the concentrations were basically evenly distributed (Figure S3).

Health risks mainly came from carcinogenic pollutants, firstly Cr, Secondly As, and Cd has the lowest risks. The average R value of Cr to a child, elder, young, and adult in the wet season was 0.0131, 0.0113, 0.0111, and 0.0124 a^−1^, respectively: while 0.0069, 0.0039, 0.0037, and 0.0048 a^−1^ in the dry season. For non-carcinogenic pollutants, Fe had the highest R value. The average R value of Fe to a child, elder, young, and adult in the wet season was 8.78 × 10^−8^, 2.17 × 10^−8^, 2.05 × 10^−8^ and 3.25 × 10^−8^ a^−1^, respectively. While 2.80 × 10^−8^, 6.91 × 10^−9^, 6.53 × 10^−9^, 1.04 × 10^−8^ a^−1^ in the dry season. The second was Mn. In the wet season, the R value of Fe and Mn accounted for more than 56% of the total, while in the dry season more than 89%. Health risks of each heavy metal to different populations are shown in Table S3.

## 4. Discussion

### 4.1. Sources of Heavy Metals

#### 4.1.1. Distribution Law and Statistical Characteristics

The study area is a typical agricultural area in China used for rice cultivation (Figure 1). According to the provincial environmental bulletin, the area of arable land in the province has increased by 725.52 hectares over the past two decades. To ensure the grain output, a large number of pesticides and fertilizers are used [32]. Additionally, the government has taken measures to improve soil fertility by increasing the content of organic and inorganic materials in the soil, which may have resulted in heavy metals entering the groundwater [33]. In addition, grain processing plants, metal processing, electroplating, tanning, and other industries are also distributed in the study area (Figure 1), resulting in the potential for related industrial wastes to enter the groundwater [32,34]. These environmental backgrounds determined the distribution of heavy metals. Among the exceeding heavy metals (As, Fe, Mn, and Ni), the VC values were less than 2, which proves that the exceeding heavy metals were widely distributed in the region. The VC value of As and Ni showed a seasonal difference, which was obviously more widely distributed in the dry season than in the wet season.

The heavy metal content of groundwater in the study area was significantly higher during the wet season than the dry season and the health risks had the same trend. Consider this phenomenon is mainly the plentiful, precipitation, surface water supplies, and the reason for the increase of groundwater [1,35]. Heavy metals produced by human pollution usually stay in the shallow unsaturated zone until precipitation directly leaches these metals into the aquifer [33]. In addition, precipitation will lead to the fluctuation of groundwater levels, during which time heavy metals in the soil of adjacent aquifers will be repeatedly leached into groundwater [36]. In the wet season, the river rises to recharge the groundwater, and the heavy metals in riverbed sediments will also enter the aquifer through the recharge [34]. Based on an environment bulletin for Heilongjiang province in 2015, the water quality of Ashley River, a tributary of Songhua River, is worse than class V. The river flows through the northern part of the study area, which is the reason for the high concentration of heavy metals in groundwater in the north.

#### 4.1.2. Sources and Genesis

Human activities can lead to changes in Cl^−^ concentration. In inland areas, the increase of Cl^−^ concentration in groundwater is often regarded as a sign of human pollution [34,37,38]. NO_3_^−^ was the three main typical pollutants in Northeast China, which was mainly from the use of pesticide and fertilizer in agricultural production [14,15,39]. The metals Cd, Cu, and Ni that were significantly related to NO_3_^−^ and Cl^−^ come from human activities. Cd in groundwater is closely related to agricultural activities. Generally, most phosphate ores contain 5–100 mg/kg of Cd [40]. In addition, the sources related to Cd might be livestock manure [41,42,43], However, they mostly stay on the soil surface and make limited contributions to groundwater [44,45], which is consistent with the statistical distribution of Cd concentration. Cu is an essential element in animals. The breeding industry often adds Cu to feed to promote animal growth, which will also lead to environmental copper pollution. The Cu in the groundwater in the study area came from this [46,47]. Ni is an important industrial raw material. Electroplating and metal smelting industries are developed in the area. The distribution of Ni was similar to these factories, which means that Ni came from industrial activities. Fe and Mn were the two other typical pollutants in the study area., which mainly from the solution filtration of iron and manganese minerals [15,21,24]. The significantly related As can also be considered as a natural source. The leaching of groundwater to rock and soil in the process of flow and exploitation leads to the high content of As, which can also be reflected in the consistency of spatial distribution. In addition, Fe and Mn also showed a negative significant correlation with NO_3_^-^ in the wet season. This is because, after the use of ammonia fertilizer, the consumption of oxygen in the nitrification process affected the release of Fe and Mn from the ore [48,49].

In the PCA analysis, in the wet season, the variance contribution rate of F1 was 26.235%, and it reveals the main source of heavy metals in groundwater in the wet season. F1 consisted of NO_3_^−^, Cl^−^, As, Ba, Fe, and Mn. Meanwhile, these metals also showed a positive correlation in the Spearman correlation analysis. According to the statistical calculation results, the contents of As, Fe, and Mn in groundwater were from a native environment. Furthermore, the release of Fe and Mn from rock and soil to groundwater is controlled by nitrogen caused by agricultural activities [24,50]. Therefore, it can be concluded that F1 mainly reflects the influence of a native environment and agricultural activities. F2 contributed the second large variance rate with a number of 20.349%, which consisted of Cd and Cu, pollutants left over from agricultural activities [51], F2 indicated that Cd and Cu in groundwater in the wet season are affected by agricultural activities. Hence, the second common factor F2 represents the influence of agricultural activities. F3 contributed a variance rate of 12.086%, which consisted of Co, Cr, Ni, and Pb. Among these metals, Cr is an important raw material in the electroplating and leather industry [52]. Pb widely exists in fossil fuels, which is a waste product of heavy industry such as metal smelting. Pb enters the aquifer with atmospheric deposition [53]. It showed that F3 mainly reflects the effect of industry activities. Finally, F4 contributed a variance rate of 10.750%, which consisted of Zn. Previous studies have shown that Zn in Songnen Plain mainly comes from the native environment [24,49]. Therefore, F4 mainly reveals the influence of a native environment.

In the dry season, the genetic types are similar to those in the wet season, but the main sources of some heavy metals have changed. The factor with the largest contribution rate of variance was F1, with a number of 25.737%, which showed the main sources of heavy metals in the dry season. The factor F1 consisted of NO_3_^-^, Cl^-^, Cd, Co, and Ni showed similar results of the Spearman correlation analysis, which were important industrial and agricultural raw materials. The sources of these heavy metals are usually related to industrial and agricultural activities under such a significant correlation [26,54]. The factor F1 mainly reflects the influence of agricultural and industrial activities. The factor F2 contributed a variance rate of 20.545%, which consisted of As and Fe. While the factor F3 was 11.819%, which consisted of Ba and Mn. Compared to the wet season, As and Fe are no longer controlled by agricultural activities. It can also be seen from the statistical data that the average concentration of Fe in the dry season is only about half of that in the wet season. This is because limited by seasonal conditions, agricultural activities in the dry season are relatively weaker, resulting in the weakening of irrigation and fertilization, which affects the release of Fe from rock and soil. On the contrary, the concentration of Ba and Mn in the dry season are small changes compared to the wet season. Therefore, the factor F2 mainly reflects the influence of a native environment, and F3 reflects the influence of a native environment and agricultural activities. The factor F4 contributed 10.653% of the variance rate. And the factor consisted of Cu, Pb, and Zn, which means the factor F4 reflects the joint influence of agricultural and industrial activities, and a native environment [3,55].

### 4.2. VS Non-Metallic Pollutants

The results of this study were compared with those of the author’s previous investigation of non-metallic health risks in Songnen Plain. Overall, heavy metals were more extensive than non-metallic pollutants in terms of pollution degree and scope. Comparison of the primary pollutants (Fe, Mn, and NO_3_-N) between studies revealed that the exceedance rate of Fe was close to NO_3_-N, while Mn was higher than NO_3_-N. The wet and dry season was 1.7 and 1.5 times that of NO_3_-N, respectively. The maximum exceedance multiples of Fe in the wet and dry season were 7.5 and 2.4 times that of NO_3_-N, respectively. And for Mn, the number was 3.4 and 2.4 times that of NO_3_-N. At the same time, heavy metals also posed stronger risks to human health. With the exception of infants in the dry season, the health risks posed by heavy metals were all higher than those of non-metallic pollutants. For non-metals, the unacceptable risk rate decreased with age, while for heavy metals, the risk increased when people are over 60 years old. The reason for this phenomenon was likely that heavy metals have stronger toxicity and cause great risks to human health once exceeding the standard [56]. The similarity between the two pollutant types was that the main source was local agricultural production for both. Therefore, the extensive use of fertilizers and pesticides should be strictly controlled.

### 4.3. Measures and Suggestions

Based on these results, reasonable suggestions that can be used as a reference in groundwater management in the study area were proposed. These suggestions cover three aspects: strengthening soil and surface water management and remediation, regional groundwater monitoring and early warnings; developing a centralized water supply; and raising people’s awareness of risk prevention.

Carcinogenic heavy metals are the main reasons affecting human health. For As and Cr, soil treatment and remediation should be enhanced so that the soil heavy metal content is in line with the local background value [27]. Relevant government agencies have established long-term and regular monitoring mechanisms, such as measuring and analyzing the content of heavy metals in the soil regularly in the dry and wet seasons so that the soil sources of heavy metals in groundwater can be effectively controlled. Surface water quality restoration should be enhanced. Heilongjiang Provincial Environmental Communique in 2019 pointed out that most of the Ashley River has been turned into IV class water, the future may be restored to the III Class of Water. The riverside water project demonstration should be promoted. Effective use of the bank filtration water system is also the key to effectively controlling the source of heavy metals in groundwater [57,58]. At present, the groundwater monitoring mechanism in the study area is imperfect, the distribution of monitoring points is limited, a regional groundwater monitoring system has not been established, and data sharing is insufficient [59,60]. Accordingly, it is necessary to establish a long-term measurement system to enable effective management of groundwater quality throughout the study area.

Non-carcinogenic heavy metals had not posed a significant threat to human health up to now. However, Fe and Mn still far exceeded standards III, which should be paid enough attention to [48,49]. For these native pollutants, controlling heavy metals pollution at groundwater sources is not sufficient to improve groundwater quality in the area [61,62]. Specifically, improvements in the process of water use by residents are needed. There are 260 administrative villages in the study area. The rural population is large, and the water used by residents is mainly obtained from private wells. As a result, the sanitary conditions of the water are not perfect, and the health risks are great. The government funds to replace the water from the self-supplied wells with a centralized water supply plant, which would effectively reduce the health risks faced by residents [63].

Finally, residents’ awareness of health risks should be raised. In addition, relevant exposure parameter manuals should be developed for local residents to obtain more reasonable health risk assessment results. Children in the study area face the greatest health risks; therefore, families can be encouraged to let children use other sources for domestic water until the health risks associated with groundwater are reduced through government policies and other measures. In addition, households can minimize the health risks associated with domestic water use by installing water purifiers [64]. Figure 3 also shows that there are certain health risks in the whole region. Therefore, a comprehensive investigation of the physical condition of people in the study area should be conducted, and a more targeted groundwater health risks prevention manual should be developed. This modeling approach is suitable for groundwater health risks when facing various heavy metals. And the mathematical statistics method provided a reliable way to analyze the sources of heavy metals in groundwater in population gathering areas with frequent industrial and agricultural activities. The results can serve as a scientific basis for local groundwater management and remediation.

## 5. Conclusions

Based on the 98 groundwater samples collected, 11 kinds of heavy metals in the groundwater in the Songnen Plain of China were analyzed. Correlation analysis and principal component analysis were used to explain the origin of these heavy metals. The HHRA model was used to assess the health risks arising from these heavy metals. In the wet season, the average concentrations of these heavy metals were as follows: Fe (2779.91 μg/L) > Mn (817.73 μg/L) > Ba (110.89 μg/L) > Zn (8.11 μg/L) > Ni (6.87 μg/L) > Co (3.23 μg/L) > As (3.12 μg/L) > Cr (2.98 μg/L) > Cu (1.75 μg/L) > Pb (0.48 μg/L) > Cd (0.05 μg/L). While in the dry season, the corresponding order is Fe (1501.62 μg/L) > Mn (609.08 μg/L) > Ba (78.53 μg/L) > Zn (7.84 μg/L) > Ni (3.85 μg/L) > Co (2.53 μg/L) > As (2.18 μg/L) > Cu (0.76 μg/L) > Pb (0.14 μg/L) > Cr (0.63 μg/L) > Cd (0.52 μg/L). As, Cr, Fe, Mn, and Ni at some points exceeded the limits. On the whole, these heavy metals were mainly distributed in the middle of the study area and along the upper reaches of the Lalin River. In the wet season, As, Ba, Fe, and Mn were related to the natural leaching and the agricultural activities; Cd and Cu were mainly from the agricultural activities; Co, Cr, Pb, and Ni were mainly from the industrial activities; Zn was mainly from the native environment. In the dry season, Cd, Cr, and Ni were related to the agricultural and industrial activities; As and Fe were mainly from the native environment; Cr was mainly from the industrial activities; Ba and Mn were related to the natural leaching and the agricultural activities; and Cu, Pb, and Zn were jointly influenced by the agricultural and industrial activities, and the native environment. The risks borne by different groups were child > elder > young > adult. Carcinogenic heavy metals were the main cause of health risks, and Cr contributed the most risks followed by As. Fe contributed the most health risks of non-carcinogenic, but it did not pose a threat to human bodies. The irrational use of pesticides and fertilizers and the discharge of industrial pollution should be controlled, and more centralized water supply wells should be constructed to enhance the pretreatment ability for ensuring the safety of drinking water. This study provides a method for the source apportionment and health risk assessment of heavy metals in complex environments, which is suitable for population gathering areas with extensive agricultural and industrial activities. The results can provide the managers with better decisions to manage groundwater.

## Figures and Tables

**Table 1:** Statistical information on the measured groundwater indicators.

Heavy Metals	Seasons	As	Ba	Co	Cr(VI)	Cu	Fe	Mn	Ni	Pb	Zn
Sta III (μg/L)		10.00	700.00	50.00	50.00	1000.00	300.00	100.00	20.00	10.00	1000.00
Min (μg/L)	Wet	0.24	41.20	0.09	1.96	0.67	8.69	1.00	0.37	0.40	1.27
	Dry	0.16	9.78	0.03	/	0.12	5.62	/	0.02	0.03	0.31
Max (μg/L)	Wet	39.78	337.60	26.32	7.81	5.93	27,434.20	4258.00	26.65	0.95	54.18
	Dry	15.63	348.29	26.64	2.36	3.10	8754.59	3205.13	24.50	0.72	54.29
Mean (μg/L)	Wet	3.12	110.89	3.23	2.98	1.75	2779.91	817.73	6.87	0.48	8.11
	Dry	2.18	78.53	2.53	0.63	0.76	1501.62	609.08	3.85	0.14	7.84
SD	Wet	5.97	68.22	6.36	1.15	1.08	5559.29	943.14	13.22	0.10	10.86
	Dry	2.98	72.22	4.82	0.65	0.61	2605.05	722.24	5.29	0.14	10.44
VC	Wet	1.91	0.62	1.97	0.39	0.62	2.00	1.15	1.92	0.22	1.34
	Dry	1.37	0.92	1.90	1.03	0.81	1.73	1.19	1.37	1.02	1.33
E (%)	Wet	4.35	0	0	0	0	39.13	71.74	8.70	0	0
	Dry	5.77	0	0	0	0	42.31	61.54	5.77	0	0
MEM	Wet	3.98	0	0	0	0	91.45	42.58	4.30	0	0
	Dry	1.56	0	0	0	0	29.18	32.05	1.23	0	0

Note:/means below detection limit; Sta III refers to the Class III of Quality Standard for Groundwater of China (GB/T 14848-2017); E means Exceedance rate; MEM means the maximum exceedance multiple.

**Table 2:** Spearman correlation analysis results of pollutants in groundwater.

Element	NO_3_^−^	Cl^−^	As	Ba	Cd	Co	Cr(VI)	Cu	Fe	Mn	Ni	Pb	Zn
Wet	season												
NO_3_^−^	1.000												
Cl^−^	0.770 **	1.000											
As	−0.263 *	−0.055	1.000										
Ba	0.219	0.354 **	0.158	1.000									
Cd	0.422 **	0.352 **	−0.629 **	0.060	1.000								
Co	0.256 *	0.256 *	−0.230	0.352 **	0.691 **	1.000							
Cr(VI)	0.132	0.252 *	0.462 **	0.166	−0.289 *	−0.143	1.000						
Cu	0.749 **	0.633 **	−0.098	0.121	0.268 *	0.101	0.264 *	1.000					
Fe	−0.542 **	−0.343 **	0.603 **	0.129	−0.374 **	−0.023	0.160	−0.513 **	1.000				
Mn	−0.342 **	0.099	0.649 **	0.142	−0.310 *	0.252 *	0.340 *	−0.277 *	0.627 **	1.000			
Ni	0.621 **	0.647 **	−0.295 *	0.328 *	0.680 **	0.735 **	0.070	0.493 **	−0.186	0.021	1.000		
Pb	0.163	0.019	−0.251 *	−0.146	0.255 *	0.195	0.068	0.244	−0.185	−0.181	0.115	1.000	
Zn	0.207	0.257 *	−0.378 **	−0.032	0.492 **	0.234	−0.224	0.232	−0.148	−0.148	0.296 *	0.409 **	1.000
Dry	season												
NO_3_^−^	1.000												
Cl^−^	0.677 **	1.000											
As	−0.317 *	−0.023	1.000										
Ba	0.238 *	0.267 *	0.123	1.000									
Cd	0.463 **	0.268 **	−0.207	0.291 *	1.000								
Co	0.150	0.283 *	0.125	0.222	0.419 **	1.000							
Cr(VI)	0.008	0.018	−0.190	−0.511 **	−0.145	−0.264	1.000						
Cu	0.605 **	0.466 **	−0.148	0.215	0.363 **	0.053	0.044	1.000					
Fe	−0.697 **	−0.337 **	0.702 **	0.067	−0.229	0.049	−0.120	−0.470 **	1.000				
Mn	−0.316 *	−0.316 *	0.612 **	0.394 **	0.026	0.388 **	−0.259	−0.145	0.634 **	1.000			
Ni	0.415 **	0.484 **	0.091	0.455 **	0.358 **	0.611 **	−0.492 **	0.084	−0.092	0.213	1.000		
Pb	0.144	0.144	0.227	0.273 *	0.359 **	−0.026	−0.211	0.417 **	−0.042	0.045	0.091	1.000	
Zn	0.154	0.154	0.057	0.169	0.181	0.180	−0.369 **	0.157	−0.102	−0.145	0.256	0.142	1.000

Note: ** represent significant at the level of 0.01, * represent significant correlation at the level of 0.05.

**Table 3:** Common factors and total variance contribution rate of pollutants.

Element	F1	F2	F3	F4	F1	F2	F3	F4
Cl^−^	**0.872**	0.161	0.148	0.239	**0.819**	−0.134	0.153	0.142
As	**0.846**	−0.184	0.172	0.275	−0.044	**0.902**	−0.064	−0.042
Ba	**0.552**	0.119	−0.091	−0.202	0.078	−0.095	**0.913**	0.000
Cd	−0.155	**0.772**	−0.298	0.318	**0.890**	−0.130	0.040	0.029
Co	0.242	0.449	**−0.684**	0.070	**0.687**	0.158	0.331	−0.133
Cr(VI)	0.420	0.220	**0.644**	−0.418	−0.275	0.117	**−0.601**	−0.241
Cu	−0.163	**0.798**	0.169	0.135	−0.004	−0.173	0.017	**0.644**
Fe	**0.775**	−0.348	−0.069	0.245	−0.080	**0.923**	0.076	−0.150
Mn	**0.880**	0.045	−0.021	−0.075	0.163	0.363	**0.780**	−0.216
Ni	0.081	−0.027	**0.764**	−0.088	**0.880**	−0.098	0.137	0.087
Pb	−0.026	0.050	**0.575**	0.260	0.051	0.263	0.154	**0.736**
Zn	0.096	0.204	0.099	**0.847**	−0.020	−0.207	−0.123	**0.544**
Total	2.886	2.238	1.329	1.182	2.831	2.260	1.300	1.172
Variance %	26.235	20.349	12.086	10.750	25.737	20.545	11.819	10.653
Cumulative %	26.235	46.584	58.670	69.419	25.737	46.282	58.100	68.754

Note: The left and right represent the wet and dry seasons, respectively. The bold numbers indicate the element coefficients that make up the main factor.

## Data Availability

Not applicable.

## Supplementary Materials

The following supporting information can be downloaded at: https://www.mdpi.com/article/10.3390/ijerph19063571/s1, Table S1: Measuring methods of parameters and detectable accuracy; Table S2: Parameters and their corresponding values in human health risks assessment; Table S3: Health risks of each heavy metal to different populations; Figure S1: Loading diagram of heavy metals in groundwater; Figure S2: Bar chart of average R value caused by carcinogenic and non-carcinogenic; Figure S3: R value of each sample.
