# Supplementary material for: Distribution, Genesis, and Human Health Risks of Groundwater Heavy Metals Impacted by the Typical Setting of Songnen Plain of NE China
Source: Int J Environ Res Public Health. 2022 Mar 17;19(6):3571. doi: 10.3390/ijerph19063571 (PMC8955772; doi:10.3390/ijerph19063571)
Supplement: Supplementary file 1 [file ijerph-19-03571-s001.zip › ijerph-1622414-supplementary.pdf]

Supplementary materials for

Distribution, genesis and human health risks of groundwater heavy metals impacted by the typical setting of Songnen Plain of NE China

Yuanzheng Zhai, Fuxin Zheng, Dongfan Li, Xinyi Cao, Yanguo Teng*

Engineering Research Center for Groundwater Pollution Control and Remediation of Ministry of Education of China, College of Water Sciences, Beijing Normal University, Beijing 100875, China; zyz@bnu.edu.cn (Y.Z.); 202131470011@mail.bnu.edu.cn (F.Z.); 201821470013@mail.bnu.edu.cn (D.L.); 202021470003@mail.bnu.edu.cn (X.C.)

* Correspondence: ygteng@bnu.edu.cn or teng1974@163. com

Supplementary Tables

Table S1 Measuring methods of parameters and detectable accuracy

Heavy metal	Measuring method	Detectable limit (μg/L)	Relative standard deviation (%)	Relative error (%)	Recovery efficiencies (%)
As	ICP-MS	0.002	2.85	1.78	91.3 - 102.2
Ba	ICP-AES	0.2	3.43	3.59	95.7 – 105.5
Cd	ICP-MS	0.0004	5.12	4.24	91.2 – 108.1
Co	ICP-MS	0.0003	6.77	2.28	88.7 – 103.5
Cr	ICP-MS	0.00008	1.48	2.20	85.5 – 107.3
Cu	ICP-MS	0.00002	2.35	2.08	96.4 – 110.5
Fe	ICP-AES	0.01	8.87	6.23	96.4 – 112.1
Mn	ICP-AES	0.5	6.46	3.33	90.7 – 107.5
Ni	ICP-MS	0.0001	2.21	3.42	93.6 – 108.5
Pb	ICP-MS	0.0003	1.05	5.21	89.4 – 112.3
Zn	ICP-MS	0.005	1.04	5.17	90.1 – 110.2

Table S2 Parameters and their corresponding values in human health risks assessment

**Risk coefficient of carcinogenic heavy metals (q_i)**			**Risk coefficient of carcinogenic heavy metals (R_{fDj})**							
As	Cd	Cr	Ba	Co	Cu	Fe	Mn	Ni	Pb	Zn
1.5	6.1	41	0.02	0.02	0.005	0.3	0.046	0.02	0.0014	0.003
**Per capita drinking water intake per unit weight (L/(kg·d))**										
Child	Young	Adult	Elder							
0.073	0.027	0.018	0.017							

Table S3 Health risks of each heavy metal to different populations

Parameter	Child		Young		Adult		Elder	
	**Wet season**	**Dry season**	**Wet season**	**Dry season**	**Wet season**	**Dry season**	**Wet season**	**Dry season**
As	4.46E-05	3.82E-05	1.66E-05	1.41E-05	1.11E-05	9.43E-06	1.05E-05	8.91E-06
Cd	2.89E-07	1.14E-07	1.07E-07	7.61E-08	7.13E-08	7.18E-08	6.73E-08	6.09E-08
Cr	0.013146	0.006913	0.012399	0.005012	0.011316	0.003992	0.01112	0.00385
Ba	5.33E-10	3.77E-10	2.08E-09	1.4E-10	1.31E-10	9.3E-11	1.24E-10	8.78E-11
Co	1.55E-10	1.19E-10	9.61E-12	4.41E-11	3.82E-11	2.94E-11	3.61E-11	2.78E-11
Cu	3.36E-10	1.45E-10	1.97E-10	5.38E-11	8.28E-11	3.59E-11	7.82E-11	3.39E-11
Fe	8.9E-09	4.81E-09	1.62E-08	1.78E-09	2.19E-09	1.19E-09	2.07E-09	1.12E-09
Mn	5.61E-09	4.18E-09	3.29E-09	1.55E-09	1.38E-09	1.03E-09	1.31E-09	9.73E-10
Ni	3.3E-10	1.81E-10	5.74E-11	6.71E-11	8.14E-11	4.47E-11	7.69E-11	4.22E-11
Pb	3.3E-10	9.31E-11	1.24E-10	3.44E-11	8.14E-11	2.3E-11	7.69E-11	2.17E-11
Zn	2.6E-11	2.46E-11	1.22E-10	9.11E-12	6.4E-12	6.07E-12	6.05E-12	5.74E-12

Supplementary Figures

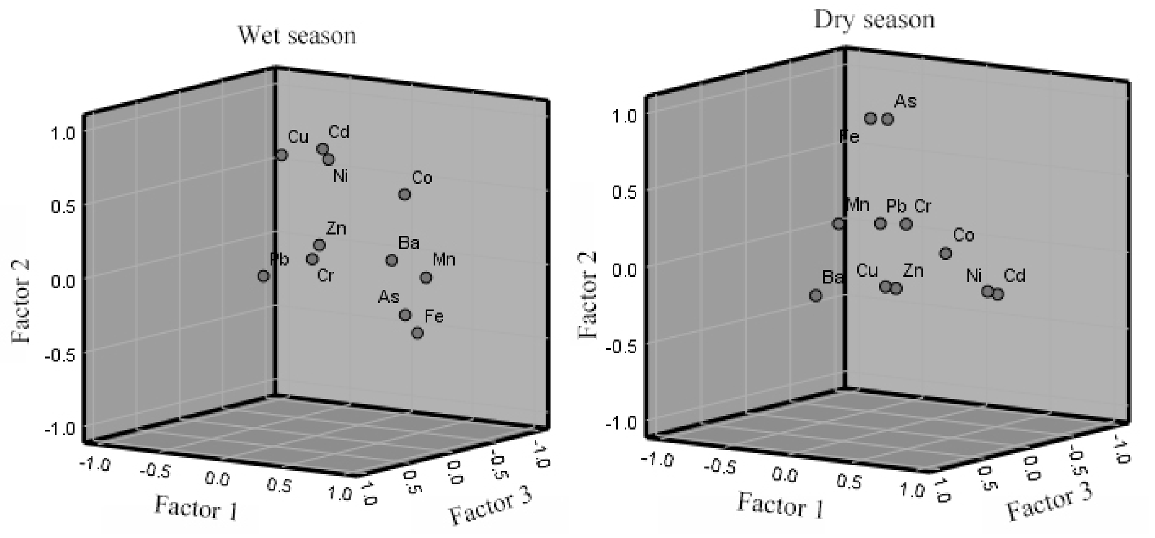

Figure S1 Loading diagram of heavy metals in groundwater

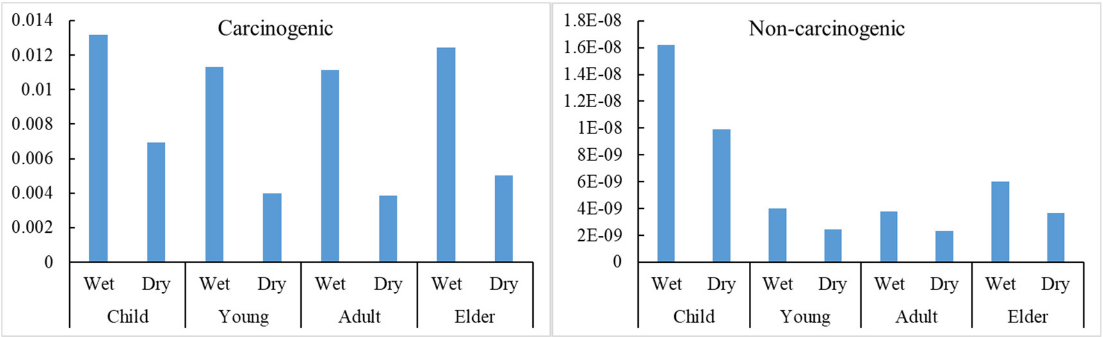

Figure S2 Bar chart of average R value caused by carcinogenic and non-carcinogenic

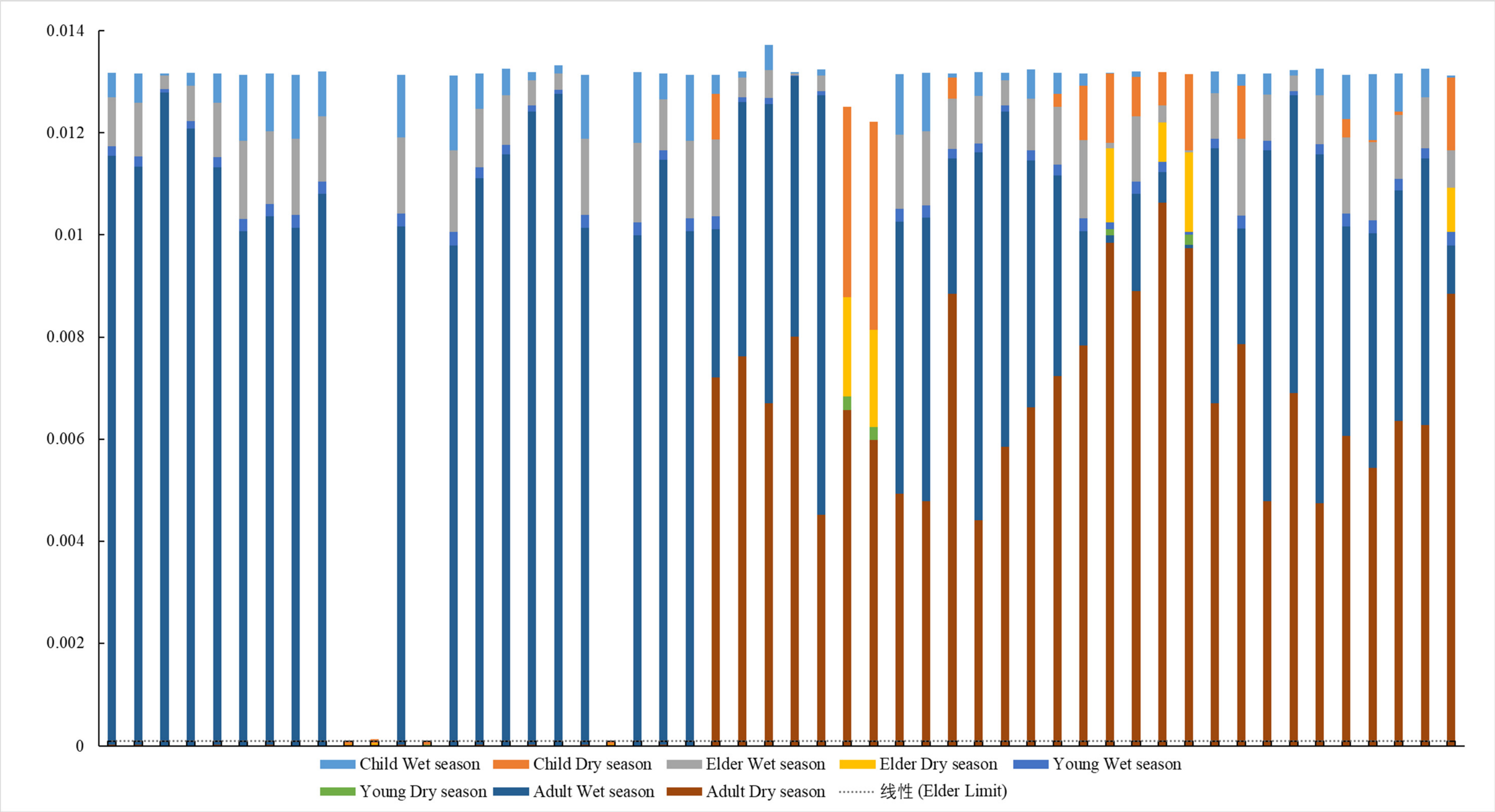

Figure S3 R value of each sample
